# The Magnitude of Occupational Class Differences in Sickness Absence: 15-Year Trends among Young and Middle-Aged Municipal Employees

**DOI:** 10.3390/ijerph14060625

**Published:** 2017-06-09

**Authors:** Hilla Sumanen, Eero Lahelma, Olli Pietiläinen, Ossi Rahkonen

**Affiliations:** Department of Public Health, University of Helsinki, P.O. Box 20 (Tukholmankatu 8B), FIN-00014 Helsinki, Finland; eero.lahelma@helsinki.fi (E.L.); olli.k.pietilainen@helsinki.fi (O.P.); ossi.rahkonen@helsinki.fi (O.R.)

**Keywords:** socioeconomic differences, relative differences, sick-leave, young adults, employees, gender

## Abstract

*Background*: Our aim was to examine the magnitude of relative occupational class differences in sickness absence (SA) days over a 15-year period among female and male municipal employees in two age-groups. *Methods*: 18–34 and 35–59-year-old employees of the City of Helsinki from 2002 to 2016 were included in our data (*n* = ~37,500 per year). Occupational class was classified into four groups. The magnitude of relative occupational class differences in SA was studied using the relative index of inequality (RII). *Results*: The relative occupational class differences were larger among older than younger employees; the largest differences were among 35–59-year-old men. Among women in both age-groups the relative class differences remained stable during 2002–2016. Among younger and older men, the differences were larger during the beginning of study period than in the end. Among women in both age-groups the RII values were between 2.19 (95% confidence intervals (CI) 1.98, 2.42) and 3.60 (95% CI 3.28, 3.95). The corresponding differences varied from 3.74 (95% CI 3.13, 4.48) to 1.68 (95% CI 1.44, 1.97) among younger and from 6.43 (95% CI 5.85, 7.06) to 3.31 (95% CI 2.98, 3.68) among older men. Conclusions: Relative occupational class differences were persistent among employees irrespective of age group and gender. Preventive measures should be started at young age.

## 1. Introduction

Occupational class is a key measure of socioeconomic position (SEP) alongside with education and income [1]. SEP refers to material and non-material resources and the social and economic factors that influence what positions individuals hold within the hierarchical societal structure [1,2]. In short, education provides knowledge, non-material resources and formal qualifications to achieve occupational class positions, which indicate status and power, and reflect material conditions related to paid work. Income provides necessary material resources and determines purchasing power, thus contributing to resources needed in maintaining good health. [1,3,4]. Usually, higher SEP means better health and health behaviors [5,6,7,8,9,10] and lower amount of SA [11,12,13,14,15].

In this article, occupational class is used as a measure of SEP as it is well suited for describing the hierarchy of the employees in the municipal workplace. Previous studies have shown that occupational class has a strong independent association with sickness absence (SA) [15,16], however the association may not always follow a clear gradient among younger employees [17,18]. Overall, occupational differences in SA tend to be large and according to previous studies larger among men than among women [15,19,20,21,22,23], but there is a lack of time trends.

As previous studies have shown, there are many factors alongside with SEP that contribute to the need and length of SA, such as gender, age, diagnosis presence of other chronic conditions, caring responsibilities, family status and deprivation [24,25,26,27,28,29,30,31,32]. However, occupational class reflects the physical and psychosocial working environment which may have an effect on the need of SA [23]. Those in the lower occupational classes tend to have more physically demanding jobs, awkward postures and monotonous movements [33] and worse physical functioning [34]. In these types of jobs, a lack of physical functional capacity may lead to the need of and prolonged SA, unless there is a possibility to evaluate the fitness to work and work safely with reduced work ability, as in the UK [29].

Those in the higher occupational classes usually have more flexibility in their jobs and thus possibly better opportunities to match their work with worsening health, for example work at home when afflicted by the flu. However, in Finland employees in higher occupational classes tend to have more mentally demanding and complex jobs [10], which may cause different exposures than physically demanding work [35]. Still, the work characteristics and access to different resources among higher occupational classes may be protective against mental illnesses [36,37]. Previous studies have found that especially physical working conditions contribute to differences in SA between occupational classes [13,15,20]. In addition, occupations where employees are under high strain but have little control over their jobs have also an increased risk of SA [38,39,40] and possible postponed return to work after SA [41,42]. Moreover, high adjustment latitude and flexible working hours promote return to work after long SA [43,44].

Previous studies have concentrated on explaining the occupational class differences in SA [13,15,19,20,21,36,45], but there is a lack of evidence on changes in the magnitude of relative occupational class differences over time among different aged employees. Generally, the relative class differences can be explained with an example that if SA days in the studied population are declining as a whole, a widening of relative difference will occur if the percentage declines in SA is larger among the higher than the lower occupational class groups [46].

SA causes significant financial and human costs, thus it is important to understand the socioeconomic patterning and its changes over time when considering efficient preventive measures. In this study we examined the magnitude of relative occupational class differences in SA days among 18–34 (younger) and 35–59-year-old (older) women and men during the period of 2002–2016.

## 2. Materials and Methods

### 2.1. Participants

Secondary data retrieved from registers are used in this study. Conventions of good scientific practice, data protection and information security have been applied. The study was based on registries and thus ethics approval was not required according to Finnish law [47].The participants in this study are employees of the City of Helsinki, Finland [48]. Helsinki is the capital of Finland and the largest (municipal) employer with approximately 40,000 employees (73% women). City of Helsinki’s personnel register was used to obtain information on gender, age, time of employment per year and job title. In this study, all permanently and temporarily employed younger, 18–34-year-old and older, 35–59-year-old female and male employees of the City of Helsinki from the years 2002–2016 were included (Table 1.). Employees with no information on job title were excluded (0.7–4.0% per year). Two age-groups were chosen based on previous knowledge on SA being differently distributed and occupational class differences being different among younger and older employees [16,17,24].

### 2.2. Sickness Absence

In this study SA days per year in employment were used as the outcome. Data on SA were collected from the employer’s SA registers. Overlapping SA spells were combined so that each SA day was counted only once. Employees of the City of Helsinki may take self-certified SA lasting 1–3 days with their supervisor’s permission. For Four to seven days of SA can be certified by qualified nurse, while SA spells lasting over week require medical certification. The SA policies are equal for all employees of the City of Helsinki, and remained similar during this study period.

### 2.3. Occupational Class

Occupational class was categorized to four hierarchical groups based on job title in the employers personnel register: managers and professionals (such as teachers and physicians), semi-professionals (such as registered nurses and foremen), routine non-manuals (such as practical nurses, child minders and clerical employees) and manual workers (such as construction workers and cleaners). Occupations in the class of managers and professionals require higher university level qualifications or are classified as managerial positions. Semi-professionals included occupations that require lower university level education such as a bachelor’s degree from a university or institution of applied sciences or are occupations that include both supervisory duties and routine tasks. Routine non-manuals and manual workers include occupations that require vocational training or no specific qualifications, and have no supervisory tasks.

### 2.4. Statistical Methods

**Figure 1:**
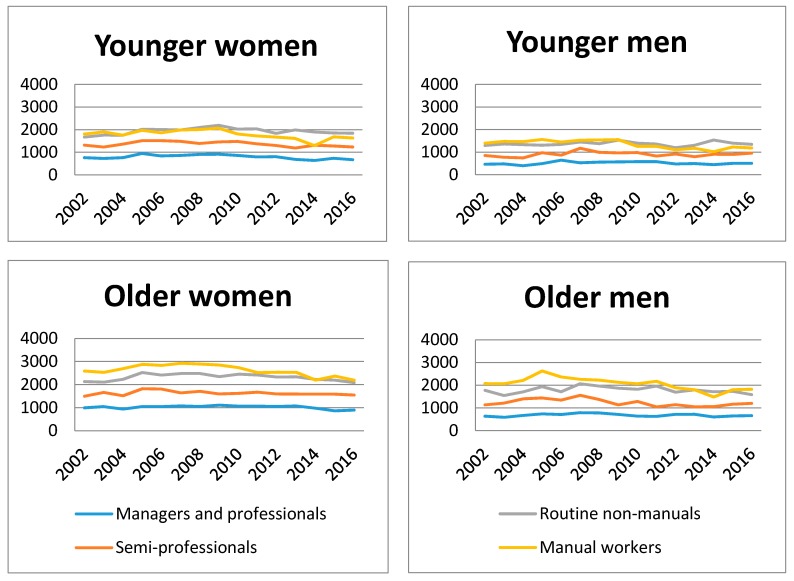
Age-adjusted sickness absence days by occupational class/100 person-years among women and men 2002–2016.

SA per 100 person-years for self-certified SA spells and days were calculated annually, i.e., each year is a cross-section for both genders and all occupational classes (Table 2). Age-adjusted SA trends are presented in Figure 1. Women and men were analyzed separately due to differences in SA levels.

The age-adjusted relative index of inequality (RII) values and their 95% confidence intervals (CI) were calculated to determine the magnitude of the relative occupational class differences in total SA days annually (i.e., each calendar year being a cross-section with regard to time) from 2002 to 2016 [49]. When calculating RII, first the values of each occupational class group were replaced with the midpoint of the cumulative proportion and then ranked between 0 and 1, Where 0 represents the highest class position and 1 the lowest class position. For example, as the managers and professionals among 18–34-year-old women in 2016 comprise 11.5% of the population, the range of young women is assigned a value of 0.0575 (0.115/2), semi-professionals comprises 30.2% of the young women, the corresponding value is 0.266 (0.115 + (0.302/2)), routine non-manuals comprises 48.9%, the corresponding value is 0.6615 (0.115 + 0.302 + (0.489/2)) and as 9,4% of the young women are manual workers, the corresponding value is 0.953 (0.115 + 0.302 + 0.489 + (0.094/2)). These values were used as continuous variables in the negative binomial regression models, and the logarithm of the time of employment was used as the offset so that different-length work contracts were taken into account. The resulting RII values can be interpreted as the rate ratio of having SA at the bottom compared to the risk at the top of the occupational class hierarchy. The RII values above 1.0 indicate higher and values below 1.0 lower SA prevalence in the lower compared to higher occupational classes. IBM SPSS statistics version 22 was used to calculate RII values.

## 3. Results

The largest occupational classes were routine non-manuals among younger and older women, and manual workers among men (Table 1). Especially the class of semi-professionals increased in size during the study period in every age and gender group, while the manual workers classes became smaller. SA days per 100 working days presented in Table 2 and age-adjusted trends in Figure 1 show that among younger employees routine non-manuals have in some years more SA than manual workers. Managers and professionals have considerably less SA than the other groups throughout the study period.

### 3.1. The Magnitude of Relative Occupational Class Differences in Sickness Absence Days among Younger Women and Men

**Figure 2:**
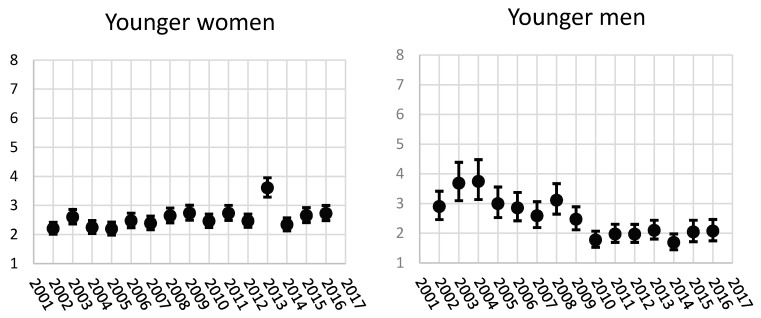
The relative index of inequality (RII) for age-adjusted sickness absence days/100 person-years according to occupational class among 18–34-year-old women and men 2002–2016.

The RII values in Figure 2 show large relative occupational class differences among younger women. These differences have remained similar (RII broadly 2.5) during the study period of 2002–2016, with an exception in 2013 (RII 3.60, CI 95% 3.28, 3.95).

Among younger men the relative class differences are also large, but there was more annual variation. From 2002 to 2009 the highest RII value was in 2004 (3.75, CI 95% 3.13, 4.48) and the lowest in 2009 (2.47, CI 95% 2.11, 2.88). Since 2010, the relative differences were lower, the RII values have been around 2, for example in 2015 2.04 (CI 95% 1.71, 2.44).

### 3.2. The Magnitude of Occupational Class Differences in Sickness Absence Days among Older Women and Men

**Figure 3:**
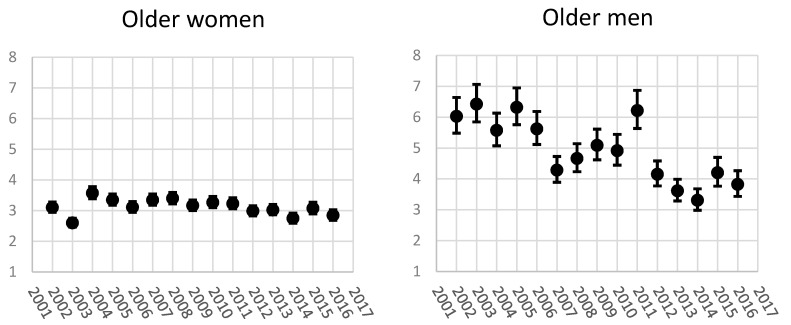
The relative index of inequality (RII) for age-adjusted sickness absence days/100 person-years according to occupational class among 35–59-year-old women and men 2002–2016.

In addition, among older women relative differences have been clear (RII around 3) (Figure 3). The largest differences were in 2004 (RII 3.56, CI 95% 3.38, 3.78) and the smallest in 2003 (RII 2.60, CI 95% 2.47, 2.75). The other years had less annual variation.

Among older men, the relative class differences were large. The RII values varied a lot from year to year. During 2002–2006 the RII values are higher, the highest value being in 2003 (6.43, CI 95% 5.85, 7.06). From 2007 to 2010 the RII values showed 4.29–5.09 times more SA days to those in the hypothetical bottom compared to those in top. In 2011 RII was significantly higher (6.22, CI 95% 5.63, 6.87) and since 2012 significantly lower, being between 3.31 (CI 95% 2.98, 3.68) in 2014 and 4.20 (CI 95% 3.76, 4.70) in 2015.

### 3.3. Differences between Groups

The magnitude of relative occupational class differences in SA days was larger among older men than any other studied group, except in 2013 the magnitudes were similar among older men and younger women. When comparing the women, the magnitude of relative occupational class differences were larger among older women than the younger women, except in 2003, 2013 and 2015–2016. When comparing older women to younger men, since 2009 older women had larger relative differences in SA. As the number of men in the data was small, the confidence intervals were wide among younger men, but in 2002–2005 the magnitude of occupational class difference was larger among younger men than among younger women. This turned since 2010 and among younger women relative occupational class differences were larger than among younger men.

## 4. Discussion

We examined the magnitude of relative occupational class differences in SA days among 18–34- and 35–59-year-old women and men from 2002 to 2016. Our main results were: (1) Clear relative occupational class differences were found over time in every studied age and gender group; (2) The magnitude of occupational class differences was larger among 35–59-year-old men than among other groups; (3) For most of the studied years, older women had larger relative differences than younger women and also larger differences than younger men since 2009; (4) Among younger age groups, men had larger relative differences during 2002–2005 than women, but this reverse for most years since 2010; (5) Among younger and older women, the relative occupational class differences were broadly stable during the study period; employees in the lowest class position had two to over three times more SA days than the in the highest class position; (6) Among younger and older men, the relative differences were larger during the beginning of the study period than in the end. Among younger men, the lowest class employees had approximately three times more SA days, and since 2010, two times more SA days than the highest class employees. Among older men, the corresponding RII values were approximately 6 at the beginning and 3.5–4 at the end of the study period.

Occupational class differences in SA are well-known [18,22,23,36,45,50] and, as expected, managers and professionals had the least amount of SA days. However, in our study, in recent years, routine non-manuals had more SA than manual workers, among younger employees in particular. Our previous study [18] examined short, 1–3 day SA among young women and the findings were similar, but in the present study the socioeconomic SA pattern was seen even among young men and in SA days per year. Further studies with different cohorts should find out if this is typical among younger employees recently.

The relative occupational class differences were largest among older men than other studied groups in this study. Our current register based data do not allow further analysis with for example diagnosis or occupational exposures, so we could only speculate the reasons. However, previous studies have reported similar findings [19,20]. Löve et al. [20] suggested that women in Sweden may have more work-related mental disorders due to stress than men, and thus more SA due to mental disorders. In addition, a recent Finnish study found out that depression-related SA was more common among women than men [51]. As SA due to mental disorders may have a reversed socioeconomic gradient due to more mentally demanding jobs being typical among higher occupational classes, this might narrow the differences. For example, Sekine et al. [10] found that mental functioning had reverse association with occupational class, i.e., those in the higher classes had worse functioning. However, this was seen only among Finnish cohort, as in British and Japanese cohorts low class employees were likely to have poorer mental functioning [10]. In addition, another study with large cohort of public sector employees in Finland found out that the gender-adjusted onset of new episodes of ≥90 day SA due to mental disorders are more common among lower occupational class positions [36], and also more evidence exist for this inverse association [37,52]. In addition, a study with survey data on middle-aged employees of the City of Helsinki showed that controlling for mental strain and job demands tended to wider occupational class differences in SA [13], thus clear conclusions for the gender difference in the studied magnitudes of class differences cannot be made based on mental work ability.

The trends shown in this study suggest that the reason for large relative differences in SA among older men might be related to older male managers and professionals having such a low amount of SA days, approximately 6 to 8 days per person per working year, whereas older male manual workers and routine non-manuals have approximately 15–26 day per year each. There are several studies that show the socioeconomic gap in healthy lifestyle among middle-aged men for example in terms of smoking [53,54], alcohol-related harm [55], physical activity [53], sports engagement [56] and poorer sleep [57], however, some variations exists by country and cohort. Self-rated health has also wide socioeconomic differences [58,59], and in Finland, Britain and Japan the occupational class differences in self-rated health are wider among men than women [59]. In addition, the male employees of the City of Helsinki had larger class differences in physical functioning than the international counterparts [59]. To add knowledge on this issue, it should be more closely examined how the differences develop over time, and if older male managers and professionals have particularly good opportunities to arrange their work so that there are no need for SA, and also, if they have more sickness presenteeism than the others.

Among men in both age-groups the relative differences were larger during the beginning than the end of the study period. There have been structural and SA changes in these groups over time, and the index used in this study responds to these changes [46]. Among older men, semi-professionals and routine non-manual classes have increased in size, while especially the amount of manual workers has decreased during the study period. In addition, the SA days among the two lowest occupational classes have decreased quite dramatically. Among younger men the two highest occupational classes have slightly increased in size and SA levels, while the SA levels among manual workers, which is a large group in size among young men, have decreased. Overall, these developments among both groups of men probably lead to decreasing relative differences in SA between lower and higher class employees.

The relative occupational class differences were more pronounced among older employees, although during the beginning of the study period young men showed larger relative differences than older women. Older employees also had more SA days than younger employees, as also noticed in other studies [60,61]. Our previous study [24] showed that the length of SA spells, which obviously affects the count days, is distributed differently among younger and older employees: the younger have more short spells and the older have more long spells. This might be related to chronic illnesses becoming more prevalent with age or that the younger employees might recover more rapidly [62,63]. Additionally, it is possible that fixed-term contracts which are more typical among the young who are just starting their working career might have an impact on SA levels, as those employees who are not permanent usually have less SA [64,65].

The larger relative differences among older than younger employees may be related to years of exposures with demanding working conditions, such as poor ergonomic conditions in manual work. In fact, the socioeconomic differences in SA due to musculoskeletal disorders are large and that type of SA is more common among older than younger employees [51]. Physical working conditions have been found to be the strongest explanatory factor for occupational class differences in SA [13,15]. In a Danish study [15] physical work environment explained more of the occupational class differences than health behavior. In a study by Hansen and Ingebritsen [19] work environment and especially ergonomic conditions were important in explaining the difference in SA between those in manual work and those in higher occupational positions. This association was strong especially among women.

Currently, it is quite common to see SA certificate as a full-time rest until recovery. In the UK, “fit note” instead of “sick note” has been associated with reduction of long SA episodes [29]. Moreover, without wider actions towards concentrating to the remaining work ability and implementing the possibility for fixed-work widely across occupations, the occupational class gap might not narrow easily, as the jobs among lower positions are currently less flexible.

The need of interventions in prevention of SA and disability is widely recognized, yet the establishment of effective programs are still lacking [66,67]. In order to reduce SA especially among those with physically or other ways demanding work, the City of Helsinki has very recently developed operational models, such as offering long-standing support to those with pain symptoms, musculoskeletal disorders or mental challenges [68,69]. However, earlier prevention where the intervention is implemented before diagnosis could be more efficient [70]. For example, in a Finnish study, age-based health check-ups offered by the occupational health care reduced subsequent SA and those in the lower occupational classes participated actively, but socioeconomic differences were not reduced [71]. If the intervention is not targeted to all employees, previous evidence shows that objective selection of the participants who could benefit for early intervention is more effective and should be promoted in future interventions [72].

### Methodological Considerations

The registers used in this study are kept by the employer and used as a base of salary payments, thus they are reliable and complete. The SA policies are same for all of the employees and have remained during the study period. However, register-based data are also a limitation, as it holds only information related to employment and not, for example information on diagnoses or lifestyle.

Our data allowed us to examine relative occupational class differences in two age groups, which showed the existing age-differences. However, among younger employees, the occupational class might not be permanent and this may have an effect to the stability of the results. However, even the youngest employees have occupational class and it is usual in municipal sector that those who are studying in some field might be working as a substitute before graduating (for example, teachers, doctors, and nurses). Thus, the final occupational class might be achieved before formal education.

The relative index of inequality takes into account the steepness of the SA differences and the actual size of the population in each socioeconomic, age and gender groups. For example, in 2013, the RII value was considerably higher level among young women than in other years. In that year, the managers and professionals group was at its largest level and had small SA amounts, whereas routine non-manuals, which are one of the two lowest occupational classes, had high amounts of SA.

Our results are obtained by examining municipal employees of a single, yet large employer. As the SA policies differ between countries and sometimes even across employment sectors, our results can be generalized with caution only to the Finnish municipal sector.

## 5. Conclusions

The magnitudes of relative occupational class differences in SA were evident among all studied groups. The class differences were larger among 35–59-year-old men than among women in respective age or among 18–34-year-old women and men. Overall, occupational class differences tended to increase from younger to older employees. The differences narrowed over time among men, but remained similar among women. The existing class differences cause notable burden of SA considering the large proportion of lower occupational classes in municipal work. As the differences were also evident among younger employees, preventive measures should be started at young age among both genders. Attention should also be paid to older employees in lower occupational classes in particular. Additionally, evaluating working conditions and other factors that support the health among higher classes and implementing those to cover employees in lower classes as well could be a possibly valuable development challenge.

## Figures and Tables

**Table 1:** Descriptive information for the study population in 2002, 2007, 2012 and 2016.

Group	Occupational Class	2002	2007	2012	2016
18–34-year-olds, *n* (%)					
Women	Managers and professionals	1067 (12.1)	1103 (13.3)	1350 (13.9)	1029 (11.5)
	Semi-professionals	2082 (23.7)	1797 (21.7)	2494 (25.7)	2692 (30.2)
	Routine non-manuals	4480 (51.0)	4307 (52.1)	4827 (49.7)	4369 (48.9)
	Manual workers	1154 (13.1)	1057 (12.8)	1032 (10.6)	836 (9.4)
	All	8783	8264	9703	8926
Men	Managers and professionals	380 (13.9)	395 (14.7)	484 (14.7)	408 (11.5)
	Semi-professionals	369 (13.5)	326 (12.1)	488 (14.8)	488 (17.8)
	Routine non-manuals	861 (31.4)	859 (32.0)	968 (29.4)	992 (36.2)
	Manual workers	1132 (41.3)	1107 (41.2)	1353 (41.1)	855 (31.2)
	All	2742	2687	3293	2743
35–59-year-olds, *n* (%)					
Women	Managers and professionals	3732 (18.2)	3715 (18.4)	3744 (19.0)	2920 (17.3)
	Semi-professionals	4478 (21.8)	5009 (24.8)	5556 (28.2)	5393 (32.0)
	Routine non-manuals	9268 (45.1)	8821 (43.6)	8352 (42.3)	7072 (42.0)
	Manual workers	3075 (15.0)	2692 (13.3)	2075 (10.5)	1455 (8.6)
	All	20553	20237	19727	16840
Men	Managers and professionals	1851 (26.5)	1771 (26.8)	1692 (26.5)	1312 (25.7)
	Semi-professionals	1142 (16.4)	1148 (17.4)	1183 (18.5)	1103 (21.6)
	Routine non-manuals	863 (12.4)	1062 (16.1)	1212 (19.0)	1224 (24.0)
	Manual workers	3127 (44.8)	2623 (39.7)	2301 (36.0)	1471 (28.8)
	All	6983	6604	6388	5110

**Table 2:** Sickness absence days/100 working-years among 18–34 and 35–59-year-old women and men in 2002, 2007, 2012 and 2016.

Group	Occupational Class	2002	2007	2012	2016
18–34-year-olds					
Women	Managers and professionals	762	853	795	674
	Semi-professionals	1314	1481	1300	1232
	Routine non-manuals	1684	1979	1845	1831
	Manual workers	1814	1959	1652	1579
	All	1498	1694	1519	1485
Men	Managers and professionals	456	520	479	507
	Semi-professionals	863	1171	906	966
	Routine non-manuals	1278	1441	1206	1331
	Manual workers	1378	1484	1096	1167
	All	1124	1274	994	1085
35–59-year-olds					
Women	Managers and professionals	977	1055	1025	888
	Semi-professionals	1458	1608	1561	1532
	Routine non-manuals	2103	2445	2287	2066
	Manual workers	2544	2869	2503	2173
	All	1813	2025	1855	1693
Men	Managers and professionals	622	770	697	646
	Semi-professionals	1109	1507	1129	1187
	Routine non-manuals	1761	2018	1669	1553
	Manual workers	2033	2215	1863	1799
	All	1440	1645	1373	1301
